# Prevalence, incidence and bothersomeness of urinary incontinence in pregnancy: a systematic review and meta-analysis

**DOI:** 10.1007/s00192-020-04636-3

**Published:** 2021-01-13

**Authors:** Heidi F. A. Moossdorff-Steinhauser, Bary C. M. Berghmans, Marc E. A. Spaanderman, Esther M. J. Bols

**Affiliations:** 1 Care and Public Health Research Institute (CAPHRI), Maastricht University, P.O. Box 616, 6200 MD Maastricht, The Netherlands; 2 Pelvic care Unit Maastricht, CAPHRI, Maastricht University Medical Centre (MUMC+), Maastricht, The Netherlands; 3 Department of Obstetrics and Gynaecology, MUMC+, Maastricht, The Netherlands

**Keywords:** Bother, Incidence, Pregnancy, Prevalence, Urinary incontinence, Systematic review

## Abstract

**Introduction and hypothesis:**

Urinary incontinence (UI) is a common and embarrassing complaint for pregnant women. Reported prevalence and incidence figures show a large range, due to varying case definitions, recruited population and study methodology. Precise prevalence and incidence figures on (bothersome) UI are of relevance for health care providers, policy makers and researchers. Therefore, we conducted a systematic review and meta-analysis to investigate the prevalence and incidence of UI in pregnancy in the general population for relevant subgroups and assessed experienced bother.

**Methods:**

All observational studies published between January 1998 and October 2018 reporting on prevalence and/or incidence of UI during pregnancy were included. All women, regardless of weeks of gestation and type of UI presented in all settings, were of interest. A random-effects model was used. Subgroup analyses were conducted by parity, trimester and subtype of UI.

**Results:**

The mean (weighted) prevalence based on 44 included studies, containing a total of 88.305 women, was 41.0% (range of 9–75%). Stress urinary incontinence (63%) is the most prevalent type of UI; 26% of the women reported daily loss, whereas 40% reported loss on a monthly basis. Bother was experienced as mild to moderate.

**Conclusions:**

UI is very prevalent and rising with the weeks of gestation in pregnancy. SUI is the most common type and in most cases it was a small amount. Bother for UI is heterogeneously assessed and experienced as mild to moderate by pregnant women.

## Introduction

Urinary incontinence (UI) is the complaint of involuntary loss of urine [1]. It is a common and embarrassing problem, evoking substantial individual morbidity, loss in quality of life and socio-economic costs [2, 3]. In addition to the loss of bladder control, the need to wear incontinence pads often harms the individuality and self-confidence of young pre-partum women [4]. UI ranges from occasionally leaking urine when coughing or sneezing [stress UI (SUI)] to UI preceded by urgency [urgency UI (UUI)], or a combination of both [mixed UI (MUI)]. In the peri-partum period women often experience UI for the first time. In general, SUI is more related to the peri-partum period, whereas the prevalence of UUI and MUI increases with age [5]. Pregnancy and (vaginal) delivery are important risk factors in the development of UI in life [2, 6]. Moreover, when SUI presents during pregnancy, the risk of having SUI at 12 years post-partum is significant [7].

The prevalence and incidence of UI in pregnancy is widely researched. However, these prevalence and/or incidence figures vary greatly throughout published reports, depending on the local setting, case definitions applied, recruited population (trimester of pregnancy and parity), and study methodology [8, 9]. Former systematic reviews focused on the prevalence of pelvic floor disorders (PFDs) among community-dwelling women [10], the prevalence of UI in nulliparous women [11] or in female athletes [12]. To our knowledge, no systematic review and meta-analysis on the prevalence and incidence of UI in pregnancy is available. Reliable prevalence and incidence rates on UI in pregnancy are not only needed to indicate the burden of the health problem, but also to better inform health professionals, policy makers and researchers to set priorities and to assist in planning management of UI[13]. Furthermore, it is known that not all pregnant women are bothered by experiencing UI. It is reported that the crude UI prevalence rate is higher and probably overestimated compared to the prevalence rate of significant or bothersome UI [3]. As bothersome UI is associated with help-seeking behaviour, this discrepancy may have crucial consequences for research planning, health care providers and policy makers [14]. However, a clear and widely accepted definition of bothersome UI still does not exist, which results in the use of heterogeneous terminology and measurement instruments.

Therefore, the primary aim of this systematic review and meta-analysis was to examine the pooled overall prevalence and incidence of UI in pregnancy in the general population, specified for relevant subcategories (trimester of pregnancy, parity, type of UI, frequency and amount). A secondary aim was to provide an overview of the measurement instruments and their outcomes for bother in relation to UI as used in included studies.

## Methods

The MOOSE statement for reporting systematic reviews and meta-analyses was followed [15]. The research protocol was published in the PROSPERO database (registration number CRD42018111991).

### Search strategy

We performed a systematic review and meta-analysis of observational studies reporting on the prevalence and/or incidence of UI during pregnancy and experienced bother in relation to UI. We searched the electronic databases of PubMed, EMBASE and CINAHL.

We used the following search terms to search all databases: pregnancy, pregn*, prepartum, pre-partum, pre partum, peripartum, peri-partum, peri partum, nulliparous, primiparous, primigrav*, primipar*, multiparous, multigrav*, multipar*, urinary incontinence, urine loss, pelvic floor disorders, pelvic floor dysfunctions, leaking urine, incontinence, prevalence, incidence, epidemiology, bothersomeness, bother* and quality of life. In the Appendix the complete search strategy for PubMed is provided. This search string was adapted for use in the other databases.

### Eligibility criteria

Observational studies published between January 1, 1998, and January 1, 2019, in Dutch, English, Portuguese, German and French were included. All studies examining prevalence and/or incidence of UI among adult primi- and multigravid women, regardless of weeks of gestation, type of UI, setting and country, were of interest. Outcomes of interest were prevalence and/or incidence of (bothersome) UI. Exclusion criteria were: articles not available in full or not reporting an overall UI prevalence of any frequency and studies examining only twin pregnancies. When articles did not report a prevalence or incidence figure or response rate, an attempt was made for estimation from the information provided. Throughout this article we use the term bother (in relation to UI) as umbrella term for related constructs [impact on daily life or quality of life (QOL)].

### Study selection

Titles and/or abstracts of studies retrieved using the search strategy and those from additional sources were screened independently by two reviewers (HM and EB) to identify studies that potentially meet the inclusion criteria. The full text of these potentially eligible studies were retrieved and independently assessed for eligibility by two reviewers. Any disagreement on eligibility was resolved through discussion with a third reviewer (BB). All the included articles were reference checked.

### Data extraction and risk of bias

Information on each study was extracted in a standardized data extraction form, based on the Cochrane Public Health Data Extraction and Assessment template[16].

To assess the risk of bias, the Joanna Briggs critical appraisal tool for studies reporting prevalence data was used [17, 18]. The checklist consists of nine questions, with the response options yes, no, unclear or not applicable. Overall risk of study bias was rated as low (defined as 8–9 criteria answered as ‘yes’), moderate (4–7 criteria answered as ‘yes’) or high risk (≤3 criteria answered as ‘yes’). The response option not applicable (occasionally scored in criteria 5) was considered to be a ‘yes’. Two reviewers extracted data independently. Inconsistencies were identified and resolved through discussion including a third author if necessary.

Characteristics regarding measurement instruments for bother were extracted in a separate standardized extraction form. The form contains items such as measurement instrument, related construct and measurement results.

### Summary measures, statistical analyses and heterogeneity

We used a random effects model to pool the inverse variance (IV) weighted prevalence of UI in individuals to avoid undue influence on the summary estimate from smaller and less precise studies or studies with a very small prevalence. Pooled prevalence and incidence values were reported with 95% confidence intervals (CI). The degree of heterogeneity was determined by the I^2^ statistic, with I^2^ > 75% labelled as considerable heterogeneity [19].

We performed subgroup analyses based on trimester, parity, type and frequency of UI, as these factors may explain why studies show varying prevalence figures. Trimesters 1, 2 and 3 were defined as weeks 1–13, 14–26 and 27 to at term (42 weeks), respectively. STATA Statistical Software, release 15, was used for analysis.

To determine the overall experienced bother in relation to UI across included studies, the total scores of the different measurement instruments for bother were converted to a (standardized) 0 to 100 scale, with 0 indicating no bother and 100 indicating extremely bothered. We classified 1 to 20 as no to mild bother, 20 to 40 as mild to moderate bother, 40 to 60 as moderate to severe, 60 to 80 as severe to very severe and 80 to 100 as extremely severe bother.

## Results

### Study selection

**Fig. 1:**
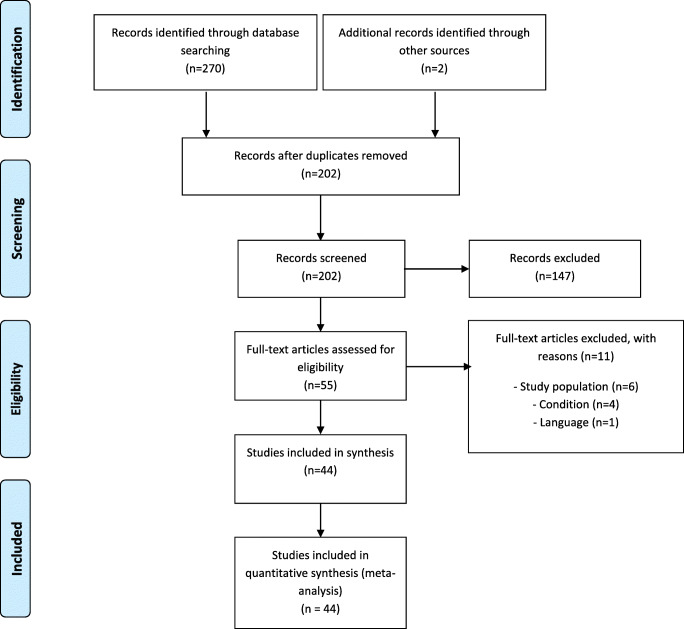
Study flow diagram

Among the 1338 papers initially identified, 44 met the eligibility criteria (Fig. 1), resulting in a total of 88,305 participants. All included studies were observational and published between 1998 and January 1, 2019.

### Risk of bias

The risk of bias items for each study are shown in Table 1. High, moderate and low risks of bias were considered to be present in 3, 34 and 7 studies respectively. Risk of bias items with the lowest ratings were 8 and 9, and risk of bias items with the highest ratings were 1 and 4.

**Table 1:** Characteristics and outcomes of included studies

Authors/year	Country	Sample	Case definition UI	Timing measurement(s)	Questionnaire validation	Mean age (y) (SD; range) ^a^	Parity(Number of children: n (%)	Sample size [response rate (%)]	Trimester of pregnancy n (%) ^a^	UI prevalence n (%) [Incidence: n (%)]	Type of UI n (%)	Risk of bias items ^**b**^
Abdullah et al. 2016 [27]	Malaysia	Nulliparous pregnant women in 3rd trimester	UI not specified	3rd trimester	Questionnaire based on ICIQ-UI-SF (Face-to-face interview by trained personnel)	< 20 y: *n* = 25 21–30 y: *n* = 254 > 30 y: *n* = 27	–	306	3rd trimester	105 (34.4)	- SUI: 68 (64.8) - UUI: 7 (6.7) - MUI: 26 (24.8) - Unknown: 4 (1.3)	2,5,9
Adaji et al. 2010 [53]	Nigeria	All pregnant women	UI not specified	All trimesters	ICIQ-UI-SF (illiterate women assisted by trained nurse)	29.6 (15–42)	- 0: 46 (22.5) - 1–4: 131 (64.2) - > 4: 27 (13.2)	204	- 1st: 9 (4.4) - 2nd: 101 (49.8) - 3rd: 93 (45.8)	43 (21.1)	- SUI: 26 (60.5) - UUI: 11 (25.6) - MUI: 4 (9.3) - Enuresis: 1 (2.3) - Unexplained: 1 (2.3)	3,5,8,9
Balik et al. 2016 [54]	Turkey	- All pregnant women - 28–40 wks gestation Exclusion criteria: - Diabetes mellitus - POP - Renal disease	UI not specified	3rd trimester	- ICIQ-UI-SF - IIQ-7 - UDI-6	29.41 (5.7; 18–44)	- 0: 90 (36) - ≥ 1: 160 (64)	250	3rd trimester $\overline{\times}$: 35.45 wks (SD 2.98; range 28–40)	93 (37.2)	- SUI: 39 (41.9) - UUI: 12 (12.9) - MUI: 42 (45.2)	3,5,7,8,9
Bekele et al. 2016 [55]	Ethiopia	- All pregnant women Exclusion criteria: - Severely sick - Kidney or urethral infection - Contra-indication for vaginal palpation	Complaint of any involuntary leakage of urine at least once during current pregnancy.	All trimesters	Questionnaire adapted	26 (16–40)	- 0: 132 (31.3) - 1–3: 243 (57.6) - ≥ 4: 47 (11.1)	422 (92.5)	- 1st: 41 (9.7) - 2nd: 101 (23.9) - 3rd: 280 (66.4)	48 (11.4)	- SUI: 28 (58) - UUI: 6 (12.5) - MUI: 12 (24.5) - Unknown: 2 (5.0)	6,7,8
Beksac et al. 2017 [56]	Turkey	- Nulliparous pregnant women - No UI before pregnancy Exclusion criteria: - Systemic disorders (DM, obesity, hypertension, urinary system problems) - Previous pelvic floor surgery	UI not specified	- 11–14 wks gestation - ± 24 wks gestation - ± 37 wks gestation	UDI-6	27.29 (3.73; 19–35)	Nullipara	61	- 11–14 wks gestation - ± 24 wks gestation - ± 37 wks gestation	- 11–14 wks: 3 (4.9) - 24 wks: 6 (9.9) - 37 wks: 16 (26.3)	- SUI: 11–14 wks: 2 (3.3), 24 wks: 4 (6.6), 37 wks: 10 (16.4) - UUI: 11–14 wks: 1 (1.6), 24 wks: 2 (3.3), 37 wks: 4 (6.6) - MUI: 11–14 wks: 0, 24 wks: 0, 37 wks: 2(3.3)	2,3,5,8,9
Bø et al. 2012 [57]	Norway	- All pregnant women - < 20 wks gestation - Living in districts - Planning to give birth at one of two study hospitals	UI not specified	28 ± 2 wks gestation	ICIQ-UI-SF	29.3 (4.9)	- 0: 351 (46.1) - ≥ 1: 410 (53.9)	772 (93.8)	$\overline{\times}$: 28 ± 2 wks gestation	310 (41.7)	- SUI: 218 (71.0) - UUI: 28 (9.1) - MUI: 36 (11.7) - Missing: 28 (8.2)	8
Brown et al. 2010 [31]	Australia	- Nulliparous pregnant women - ≥ 18 y - ≤ 24 wks gestation	Leakage of urine at least once per month	- ≤ 24 wks gestation - 31 wks gestation	- Validated questionnaire (≤ 24 wks gestation-questionnaire) - 31 wks gestation- computer assisted telephone interviews) - Sandvik questionnaire	- 18–24: *n* = 213 (14.1%) - 25–29: *n* = 430 (28.5%) - 30–34: *n* = 583 (38.7%) - ≥ 35: *n* = 281 (18.6%)	Nullipara	- ≤ 24 wks gestation: 1507 (22.0) - 31 wks gestation: 1454	- ≤ 24 wks: $\overline{\times}$: 15.03 (SD 3.09, range 6–24) - 31 wks: $\overline{\times}$: 31 (SD 0.03; range 27–38)	- ≤ 24 wks: 256 (17.0) - 31 wks: 813 (55.9) [Incidence: - ≤ 24 wks: 146 (16.4) - 31 wks: 561 (63.2)]	- SUI: ≤ 24 wks: 125 (8.3), 31 wks: 536 (36.9) - UUI: ≤ 24 wks: 38 (2.5), 31 wks: 86 (5.9) - MUI: ≤ 24 wks: 93 (6.2), 31 wks: 191 (13.1)	8,9
Chan et al. 2013 [58]	China	- Nulliparous pregnant women - ≥ 18 y - Singleton pregnancy - Chinese - No UI before pregnancy	Presence of either SUI or UUI	1st, 2nd, 3rd trimester	PFDI	30.6 (3.8)	Nullipara	328 (74.2)	All trimesters: 328 (100)	- 1st: 38 (11.5) - 2nd: 112 (34.1) - 3rd: 134 (41.8)	- SUI: 1st: 30 (9.1), 2nd: 106 (32.35), 3rd: 124 (37.8) - UUI: 1st: 16 (4.9), 2nd: 17 (5.2), 3rd: 47(14.3) - MUI: 1st: 8 (2.4), 2nd: 11 (3.3), 3rd: 34 (10.4)	–
Daly et al. 2018 [32]	Ireland	- Nulliparous pregnant women - ≥ 18 y - ≤ 24 wks gestation	Reporting any leakage	During pregnancy (< 24 wks)	- Questionnaire, adapted from existing valid questionnaire - Sandvik questionnaire	- 18–24: *n* = 81 (9.4%) - 25–29: *n* = 205 (23.8%) - 30–34: *n* = 357 (41.5%) - 35–39: *n* = 189 (22.0) - ≥ 40: n = 28 (3.3%)	Nullipara	860 (46.7)	< 24 wks	330 (38.7) [Incidence: 120 (21.7)]	- SUI: 147 - UUI: 40 - MUI: 128 Incidence: - SUI: 71 - UUI: 9 - MUI: 33	8
De Oliveira et al. 2013 [43]	Brazil	- All pregnant women - Singleton pregnancy Exclusion criteria: - Clin. or obst. Interference during pregnancy - UTI or renal inf. Last 4 wks - Neurological diseases - Cognitive deficit - Illiterate women - Premature birth - Absence of prenatal care	Women reporting not having any UI were defined as continent. Women who reported symptoms of incontinence were defined as “incontinent”.	3rd trimester: last four weeks of pregnancy	- ICIQ-UI-SF (interview)	27	- 1: 200 (40.4) - 2–3: 206 (41.6) - ≥ 4: 89 (18.0)	495	3rd trimester	352 (71%)	–	5,7,8,9
Dinc et al. 2018 [59]	Turkey	- All pregnant women - Any gestational age Exclusion criteria: - 1 UTI - History of urological/ gynaecological surgery	UI not specified	All trimesters	Self-developed questionnaire (face-to-face interviews)	26.18 (5.07; 17–42)	- 0: 393 (52.4) - 1: 248 (33.1) - > 1: 107 (14.3) - Missing n = 2	750	- 1st: 128 (17.1) - 2nd: 311 (41.45) - 3rd: 311 (41.45)	300 (40.0) By trimester: 1: 38 (29.7) 2: 100 (32.2) 3: 162 (52.1)	SUI: 241 (80.3) UUI: 12 (4.0) MUI: 47 (15.7)	5,8,9
Dolan et al. 2004 [60]	UK	- Nullipara	Any UI within last 3 months	34–40 wks gestation	- Self-developed urinary incontinence questionnaire - KHQ - 34–40 wks: interview, other postal	26 (5.26)	Nullipara	492	34–40 wks	- Antenatal: 175 (35.6) - Pre-pregn: 17 (3.5)	- SUI: antenatal: 75 (49.0) - UUI: antenatal: 4 (2.6) - MUI: antenatal: 74 (48.4) - Unexplained:22	6,7,8,9
Groutz et al. 1999 [61]	Israel	- Nulliparous, primiparous and grand multiparous women	Stress UI: involuntary leakage of urine with coughing, laughing, sneezing, or any other physical effort	2nd or 3rd day post-partum	Interview	20–43	0: 100 1: 100 ≥ 5: 100	300	1–40 wks	127 (42.3) By parity: - 1: 28 (22.0) - 2: 49 (38.6) - ≥ 5: 50 (39.4)	–	3,4,5,6,8,9
Hansen et al. 2012 [62]	Denmark	- Primipara - ≥ 18 y	Any urinary leakage	UI during pregnancy (last 3 months)	- ICIQ-UI-SF - Interference daily life	28.2 (4.8)	Nullipara	1018 (63)	3rd trimester	327 (32.1)	- SUI: 243 (23.9) - UUI: 31 (3.0) - MUI: 53 (5.2)	8
Herath et al. 2017 [21]	Sri Lanka	- Nulliparous pregnant women - Second and third trimester Exclusion criteria: - Condition which prohibits physical exercise	UI not specified	2nd and 3rd trimester	Not validated questionnaire (interviewer-administered questionnaire)	26.4 (4.4; 18–43)	Nullipara	1017	- 2nd trim: 189 (18.6) - 3rd trim: 828 (81.4)	- 192 (18.9)	- SUI: 94 (9.2) - UUI: 79 (7.8) - MUI: 19 (1.9)	9
Højberg et al. 1999 [28]	Denmark	- All pregnant women - 16 wks of gestation	Involuntary loss of urine within the last year	16 wks of gestation	- Self-developed questionnaire	15–24: *n* = 1347 (17%) 25–29: *n* = 3253 (42%) 30–34: *n* = 2365 (30%) ≥ 35: *n* = 830 (11%)	- 0: 4103 (53) - 1: 2643 (34) - 2: 851 (11) - ≥ 3: 198 (2)	7795 (97)	16 wks	693 (8.9) By parity: - 0: 136 (3.9) - 1: 361 (13.8) - ≥ 2: 169 (16.2)	- SUI: 385 (55.2) - UUI: 53 (7.6) - MUI: 185 (26.6) - Unclassified: 17 (0.2) - Missing *n* = 53	8
Huebner et al. 2010 [63]	Germany	- Primipara - Singleton pregnancy - Cephalic presentation - Vaginal delivery - Duration of pregnancy ≥ 38 wks gestation	UI not specified	1st & 2nd half of pregnancy	Self-developed questionnaire	28.1 (4.7)	Nullipara	411 (67.4)	–	- 1st half pregnancy: 15 (3.6) - 2nd half pregnancy: 108 (26.3)	–	5,6,8
Hvidman et al. 2002 [20]	Denmark	- Nulli- and primiparous women	UI not specified	During 1st and 2nd pregnancy	- Not validated questionnaire	28.1	- 0: 352 (54.9) - 1: 290 (45.1)	642 (60.3)	- 1st: 27 (4.2) - 2nd: 61 (9.5) - 3rd: 133 (20.7)	- Nulliparous: 70 (19.9) - Primiparous: 72 (24.8) [Incidence: - Nulliparous: 59 (16.8) Primiparous: 24 (8.4)]	–	5,6,8
Jean-Michel et al. 2018 [42]	USA	- Pregnant women ≤ 25 y - Presenting to the labor and delivery triage unit for routine obstetric care or admitted to the maternity ward. Exclusion criteria: - < 12 wks gestation	UI not specified	3 mths preceding study enrollment	3IQ	20.3 (2.6)	- 0: 63 (64) - ≥ 1: 35 (36)	98 (82.4)	$\overline{\times}$: 34.5 wks (SD 7.5) - 1st: 1 - 2nd: 18 - 3rd: 79	51 (52.0)	- SUI: 32 (63) - UUI: 16 (31) - MUI: 0 - Unclassified: 3 (6)	3,5,6,8
Kocaöz et al. 2010 [64]	Turkey	All pregnant women	UI not specified		- ICIQ-UI-SF - Wagner’s QoL scale (Face-to-face interviews)	28.1 (1.29)	- 0: 175 (44.5) - 1: 150 (52.3) - 2: 53 (16.5) - 3: 10 (3.4) - ≥ 4: 5 (1.7)	393	- 1st: 27 (6.9) - 2nd: 47 (11.9) - 3rd: 319 (81.2)	106 (26.97) By trimester: - 1st: 3 (11.1) - 2nd: 9 (19.1) - 3rd: 94 (29.5)	- SUI: 58 (54.7) - UUI: 27 (25.5) - MUI: 17 (16.0) - Unclassified: 4 (3.8)	5,8,9
Kok et al. 2016 [65]	Turkey	- All pregnant women - ≥ 18 y Exclusion criteria: - High risk pregnancy	All responses other than ‘never’ on the questions ‘how often do you leak urine?’ and ‘How much urine do you usually leak?’	Any gestational age	- ICIQ-UI-SF - I-QoL	30.2 (4.44) - ≤ 29: *n* = 129 (44.9%) - ≥ 30: *n* = 158 (55.1%)	- 0: 112 (39.0) - ≥ 1: 127 (44.3) - Missing: 48 (16.7)	287	$\overline{\times}$: 29.5 wks (SD 8.29)	61 (21.3) By trimester: - 1st: 2 (3.3) - 2nd: 5 (8.2) - 3rd: 54 (88.2)	- SUI: 44 (72.1) - UUI: 16 (26.1) - MUI: 1 (1.8)	2,3,7,8,9
Liang et al. 2012 [66]	Taiwan	- Nullipara - ≥ 36 wks pregnancy delivered - Singleton pregnancy Exclusion criteria: - Severe cardiopulmonary or renal diseases - Preeclampsia - Insulin dependent DM - Neurogenic diseases - Surgery for POP or UI	Experiencing leakage of urine at least once a month	2nd or 3rd day post-partum	Liang et al. LUTS questionnaire (face-to-face interview)	29.4 (4.1)	Nullipara	1501	1st -3rd	563 (37.5)	- SUI: 1st trim: 147 (9.8), 2nd trim: 208 (13.9), 3rd trim: 400 (26.7) - UUI: 1st trim: 16 (1.1), 2nd trim: 33 (2.2), 3rd trim: 71 (4.7) - MUI: 1st trim: 32 (2.1), 2nd trim: 46 (3.1), 3rd trim: 91 (6.1)	5,8,9
Lin et al. 2018 [8]	Taiwan	- All women delivering after 28 wks gestation Exclusion criteria: - Multiple gestation - Severe cardio-pulmonary or renal diseases - Preeclampsia - Insulin dependent diabetes mellitus - Neurogenic diseases - UI before pregnancy	SUI; losing urine on coughing, sneezing, or physical exertion	During pregnancy	- Interview in obstetric ward - LUTS questionnaire - SF-12 - IIQ-7 - UDI-6	32.6 (4.3)	$\overline{\times}$: 1.7 (SD: 0.7)	866	1st -3rd	446 (51.5)	–	8,9
Luo et al. 2017 [67]	China	- All pregnant women - Singleton pregnancy Exclusion criteria: - UI before pregnancy - Abdominal and vaginal surgery - DM - Hypertension - Placenta previa - Threatened abortion - Amniotic fluid abnormalities -Foetal growth restriction - Vaginal bleeding Exclusion criteria: - Failure to complete all investigation content and unreliable pelvic ultrasound data	UI not specified	Any gestational age	ICIQ-UI-SF	30.5 Nullipara: 28.4 (3.8) Para: 32.7 (3.70)	- 0: 179 (52.3) - ≥ 1: 163 (47.7)	342	Nullipara: - 1st: 64 (35.8) - 2nd: 60 (33.5) - 3rd: 55 (30.7) Para: - 1st: 58 (35.6) - 2nd: 57 (35.0) - 3rd: 48 (29.4)	135 (39.5)	–	3,8,9
Mallah et al. 2014 [68]	Iran	- Vaginal delivery - Nullipara - Healthy women Exclusion criteria: - UI before pregnancy - History of pelvic trauma, anus and rectal surgery - Previous urinary/ gastrointestinal tract impairment	UI not specified	1st - 3rd trimester	- Self-developed questionnaire - VAS severity	28.1 (3.7, 19–32)	Nullipara	441	–	- 1st trim: 44 - 2nd trim: 85 - 3rd trim: 194	–	2,5,6,7,8,9
Marshall et al. 1998 [69]	UK	All women 2 or 3 days past delivery	UI not specified	2nd or 3rd day post-partum	Self constructed questionnaire	–	- 0: 3562(45.9) - 2–4: 3826 (49.3) - ≥ 5: 374 (4.8)	7762	1st -3rd	3570 By parity: - 0: 1755 -2–4: 1683 - ≥ 5: 132	–	4,6,8,9
Martin-Martin et al. 2014 [70]	Spain	- Pregnant women - Singleton pregnancy Exclusion criteria: - UI before pregnancy	UI not specified	3rd trimester	- (Modified) ICIQ-UI-SF (questionnaire given end of 3rd trimester)	31 (5.1)	- 0: 224 (58.8) - ≥ 1: 160 (41.9)	381 (82.6)	3rd trimester	118 (31)	- SUI: 84 (71%) - UUI: 4 (3.4%) - MUI: 25 (21.5%) - Unclassified: 5 (4.3%)	6,7,8
Martinez Franco et al. 2014 [71]	Spain	- Pregnant women - < 13 wks gestation - > 28 wks gestation	UI not specified	1rd and 3rd trimester	- ICIQ-UI-SF - PFDI-20	30.8 (16–42)	- 0: 122 (54.5) - 1: 91 (40.6) - 2: 8 (3.6) - 3: 3 (1.3)	224	- 1st: 58 (25.9) - 3rd: 166 (74.1)	77 (34.4) By trimester: - 1st: 11 (19.0) - 3rd: 66 (39.8)	- SUI: 37 (48) - UUI: 7 (9.1) - MUI: 25 (32.5) - Unclassified: 8 (10.4)	3,5,8,9
Martins et al. 2010 [72]	Brazil	-Pregnant women Exclusion criteria: - High-risk pregnancy - Parents who did not give consent to pregnant women < 18 y - Prenatal care at private clinics	UI regardless of amount-	Any gestational age	- Questionnaire designed and validated for this research (interview)	24.26 (14–45)	- 0: 248 (49.6) - 1: 133 (26.6) - 2: 67 (13.4) - 3: 32 (6.4) - ≥ 4: 20 (4.0)	500 (100)	All trimesters	319 (63.3)	- SUI: 196 (39.2) - UUI: 201 (40.2)	8
Mørkved et al. 1999 [73]	Norway	- All women who delivered at hospital	UI not specified	- Before pregn. - During pregn.	Structured interview 8 wks post-partum	28 (19–40)	$\overline{\times}$: 1.8 (1–5) - 1: 52 (36.1) - 2: 54 (37.5) - 3: 31 (21.5) - ≥ 4: 7 (4.9)	144 (72)	All trimesters	60 (42) By parity: - 1: 18 (35) - 2: 20 (37) - 3: 17 (55) - ≥ 4: 5 (70)	–	3,8
Nigam et al. 2016 [29]	India	- All pregnant women - > 34 wks pregnancy Exclusion criteria: - High risk pregnancy	UI not specified	34 wks gestation	- Questionnaire (based on ICIQ-UI-SF) - Interview	- ≤ 20: n = 19 - 21–25: *n* = 194 - 26–30: *n* = 162 - 31–35: n = 25	- 0: 153 (38.25) - ≥ 1: 247 (61.75)	400	Late 3rd trim	301 (75.3)	- SUI: 219 (72.7) - UUI: 16 (4.0) - MUI: 66 (21.9)	7,8,9
Okunola et al. 2018 [25]	Nigeria	- Pregnant women - 18–45 y Exclusion criteria: - DM - UI prior to pregnancy - UTI - Use of parasympathomimmetic or sympatholytic drug - Urological/ gynaecological surgery	UI not specified	Any gestational age	- ICIQ-UI-SF - Illiterate participants were assisted by 2 trained nurses	29.9 (5.3)	- 0: 150 (33.9) - ≥ 1: 292 (66.1)	442 (85.5)	- 1st: 12 (2.7) - 2nd: 83 (18.8) - 3rd: 47 (78.5)	124 (28.1) By parity: - 0: 43 (28.7) - ≥ 1: 81 (27.7)	- SUI: 77 (17.4) - UUI: 30 (6.8) - MUI: 17 (3.9)	5,8
Raza-Khan et al. 2006 [34]	USA	- All pregnant women Exclusion criteria: - Maternal history of preexisting DM - Active cardiac disease excl. Mitral valve prolapse - Neurological disease - Urinary tract surgery - Congenital genito-urinary abnormalities	Any MESA answer of ‘sometimes’ or ‘often’	3rd trimester	- MESA - Hunskaar Severity Index	28 (17–41)	- 0: 37 (32.7) - 1: 47 (41.6) - 2: 18 (15.9) - 3: 10 (8.8) - 4: 1 (0.9)	113	3rd trimester: $\overline{\times}$: 34.8 (2.6)	83 (74)	- SUI: 36 (32) - UUI: 4 (4) - MUI: 42 (37) - Unclassified: n = 1	3,8,9
Rocha et al. 2017 [74]	Portugal	- ≥ 18 y - Singleton pregnancy Exclusion criteria: - (Para)sympatica DM - UI before pregnancy - UTI/ genital infections - Urogynaecological surgery - Actual(this pregnancy)pre-term labor - Foetal death	UI not specified	Directly post-partum	Adapted ICIQ-UI-SF	29.3 (5.7)	- 0: 108 (45.6) - 1: 126 (53.2) - ≥ 2: 3 (1.3	237	3rd trimester	123	- SUI: 101 (82.1) - UUI: 12 (10) - MUI: 10 (7.9)	3,8,9
Rogers et al. 2017 [75]	USA	- Pregnant > 18 y - Nulliparous - Singleton foetus - No serious medical problem - < 36 wks gestation	UI score > 0 on ISI	1rd - 3rd trimester	- ISI - QUID - IIQ-7	24.2 (5.1)	Nullipara	623	- 1st: 124 (19.9) - 2nd: 403 (64.7) - 3rd: 96 (15.4)	- 1st: 40 (33) - 2nd: 176 (44) - 3rd: 407 (69)	SUI - 1st: 10 (25) - 2nd: 60 (34) - 3rd: 135 (34) UUI - 1st: 19 (48) - 2nd: 81 (47) - 3rd: 194 (48)	2,5,8,9
Sharma et al. 2009 [33]	India	- All pregnant women	UI not specified	1st - 3rd trimester	Questionnaire designed for this study (interview)	26.5 (18–39)	- 0: 80 (33.3) - 1–3: 133 (55.4) - 4–8: 27 (11.3)	240	- 1st: 29 (12.1) - 2nd: 89 (37.8) - 3rd: 122 (50.1)	62 (25.8) By trimester: - 1st: 7 (24.1) 2nd: 21 (23.6) 3rd: 34 (27.9)	- SUI: 46 (19.2) - UUI: 7 (2.9) - MUI: 9 (3.8)	2,3,5,7,8
Solans-Domènech et al. 2010 [26]	Spain	- Healthy nulliparous pregnant women Exclusion criteria: - UI before pregnancy - Neurological disease - Cognitive disorders - Urological pathology(non infectious) - Abortion - Impaired mobility - Previous urogynaecologic surgery - Current treatment with drugs (benzodiazepines, diuretics)	UI not specified	1st - 3rd trimester	- ICIQ-UI-SF - ISI	–	Nullipara	1128	1st - 3rd trimester	441 (39.1) By trimester: - 1st: 84 (8.3) - 2nd: 319 (31.8) - 3rd: 322 (28.5) [Incidence: 441 (39.1) By trimester: - 1st: 84 (8.3) - 2nd: 282 (28.1) - 3rd: 141 (15.2)]	- SUI: 1st trim: 57 (67.9), 2nd trim: 250 (79.8), 3rd trim: 255 (79.2) - UUI: 1st trim: 15 (17.9), 2nd trim: 25 (7.8), 3rd trim: 22 (6.8) - MUI: 1st trim: 3 (3.6), 2nd trim: 20 (6.3), 3rd trim: 18 (5.6) - Unclassified: 1st: 9 (10.7), 2nd trim: 20 (6.3), 3rd trim: 22 (6.8)	9
Sottner et al. 2006 [76]	Czech Republic	Nullipara	UI not specified	1rd - 3rd trimester (questions asked 2nd day after delivery)	Questionnaire designed for this study	–	Nullipara	339 (71.5)	All trimesters *(each trimester same sample)*	- 1st: 56 (16.5) - 2nd: 118 (34.8) - 3rd: 218 (64.3)	- SUI: 177 (52.2) - UUI: 115 (33.9) Before pregnancy: - SUI: 52 (15.3) - UUI: 18 (5.3)	4,6,8
Spellacy et al. 2001 [44]	USA	- Healthy pregnant women - ≥ 18 y	UI not specified	3rd trimester	Self-developed questionnaire (3rd trim: face to face Interview)	25.4 (5.34)	0–4	50	3rd trimester	31 (62)	–	2,3,6,7,8,9
Tanawattanacharoen et al. 2013 [45]	Thailand	- Pregnant women Exclusion criteria: - Multiple pregnancy - UI before pregnancy - Urethra/bladder surgery - LUTS and infection during pregnancy - DM - Hypertension with diuretic treatment - Incomplete medical record	UI not specified	≥ 36 wks gestation	Modified ICIQ-UI-SF	- < 20: *n* = 17 (4.1%) - 20–29: *n* = 222 (53.8%) - 30–39: *n* = 165 (40.0%) - 40–49: *n* = 9 (2.2%)	- 0: 182 (44.1) - ≥ 1: 231 (55.9)	413	≥ 36 wks	222 (53.8)	- SUI: 221 (53.5) - UUI: 83 (20.0) - MUI: 82 (19.8)	6,8,9
Thomason et al. 2007 [46]	USA	- Primipara Exclusion criteria: - Genital anomalies - Diabetes with risk of UTI - Prior urinary tract infection/surgery - Pregnant - Delivered by CS - UI before pregnancy	Have you involuntarily lost or leaked any amount of urine?	≥ 35 wks gestation (questions asked 6–9 months post-partum)	Self-constructed questionnaire	- Primiparous continent: 29.7 - Primiparous incontinent: 29.8	Nullipara	121 (75.6)	≥ 35 wks gestation	62 (51)		3,8
Valeton et al. 2011 [77]	Brazil	- Pregnant women - ≥ 28 wks gestation - 14–30 y Exclusion criteria: - UTI/gynaecological infection - Urogynecology surgery - Use of parasympa-thomimetic/sympatholytic drugs - DM - Premature labor in current pregnancy - Foetal death - Delivered infant in another institution	Symptoms of involuntary loss of urine	3rd trimester	- Self-reported UI - KHQ	23.2 (3.69)	- 0: 177 (51.6) - 2: 105 (30.6) - ≥ 3: 61 (17.8)	343	3rd trimester	105 (30.6) By parity: - 0: 89 (26) - 2: 103 (30) - ≥ 3: 151 (44)		7,8,9
Wesnes et al. 2007 [22]	Norway	All pregnant women	Everybody answering yes on the entry questions regarding UI before or during pregnancy or answering affirmatively about frequency, amount or volume before or during pregnancy, were defined as incontinent.	30 wks (3rd trimester)	Questionnaire	29.5 (14–47)	- 0: 19.981 (46.2) - ≥ 1: 23.298 (53.8)	43.279 (45)	3rd trimester	25.121 (58.1) [Incidence: 13.978 (45.6) SUI: 9.634 (31.5) UUI: 1.231 (4.0) MUI: 3.113 (10.2)]	- SUI: 15.961 (36.9) - UUI: 2.083 (4.8) - MUI: 7.077 (16.4)	8
Zhu et al. 2012 [78]	China	- Primipara - ≥ 28 wks gest.	UI not specified	- 37–42 wks gestation	BFLUTS	26.4 (4.0)	Primiparous	10.098	Late pregnancy	2.696 (26.7)	- SUI: 1.878 (18.6) - UUI: 202 (2.0) - MUI: 434 (4.3) - Other UI: 182 (1.8)	5,8,9

^a^ Unless otherwise stated

^b^ Each number represents the items where risk of bias exists (based on the Joanna Briggs critical appraisal tool [17]. 1 = Was the sample frame appropriate to address the target population? 2 = Were study participants sampled in an appropriate way? 3 = Was the sample size adequate? 4 = Were the study subjects and the setting described in detail? 5 = Was the data analysis conducted with sufficient coverage of the identified sample? 6 = Were valid methods used for the identification of the condition? 7 = Was the condition measured in a standard, reliable way for all participants? 8 = Was there appropriate statistical analysis? 9 = Was the response rate adequate, and if not, was the low response rate managed appropriately?

UI = urinary incontinence, SUI = stress urinary incontinence, UUI = urgency urinary incontinence, MUI = mixed urinary incontinence, ICIQ-UI-SF = International Consultation on Incontinence Questionnaire-Urinary Incontinence Short Form, wks = weeks, POP = pelvic organ prolapse, IIQ-7 = Incontinence Impact Questionnaire, UDI-6 = urogenital distress inventory, DM = diabetes mellitus, y = years, clin = clinical, Obst = obstetric, UTI = urinary tract infection, inf = infection, QoL = quality of life, I-QoL = incontinence quality of life, PFDI-20 = pelvic floor distress inventory, ISI = Incontinence severity index, QUID = Questionnaire for urinary diagnosis, 3-IQ = 3 incontinence questionnaire, mths = months, PFDI = Pelvic Floor Distress Inventory, KHQ = Kings Health Questionnaire, sec = second, trim = trimester, MESA = Medical, Epidemiological, and Social Aspects of Ageing Questionnaire, BFLUTS = Bristol Female Lower Urinary Tract Symptom questionnaire

### Study characteristics

Seventeen studies originated from Asia, 15 from Europe, 8 from the USA, 3 from Africa and 1 from Oceania. The majority of women were included from a (tertiary) hospital. Other studies included women from a civil registration system [20], midwifery area [21], hospital and maternity unit [22] or obstetric/child health clinic [23, 24]. Table 1 summarizes the study characteristics of included studies.

Thirteen studies reported on (measurement instruments for) bother, whereas one study (73) reported on two measurement instruments. The result of only one measurement instrument was reported for this study, as the second one (SF-36) was incomplete. Table 2 provides an overview of the measurement instruments as used in included studies, with the original and the converted (0–100 scale) measurement results.

**Table 2:** Measurement of bother and results

Measurement instrument	Background information on measurement instrument	Study	Original measurement result (mean)	Trimester/ weeks	(Converted) measurement results (0–100)
ICIQ-UI-SF (0–21)	To assess symptoms of UI and impact on QoL. (4 questions, question 4 is on moment of UI and is not within the calculation of the total).	54	4.1	AT	19.3
		58	6.3	(28 wks ± 2 wks) T3	30.0
		44	12.1	(last 4 wks pregn) T3	57.6
		63	6.2	T3	29.5
		73	6.6	(T1 and T3) AT	31.4
		26, 27	Results reported in categories. No total score		
ICIQ-UI SF Question 3 (QoL) (0–10)	Question 3 of the ICIQ-UI SF is on the interference in daily life of UI	69	Nulliparous 2.7	AT	26.7
			Multiparous 3.6	AT	35.8
			3.1	AT	31.3
		72	3.5	T3	34.8
I-QOL	Quality of life in persons with UI. 3 subscales: 1. Avoidance and limiting behaviour, 2. Psychosocial impact, 3. Social embarrassment. (22 questions)	67	82.4	AT	17.7
IIQ	Interference of UI of women’s daily life and the bothersomeness. 4 subscales: 1. Physical activity, 2. Travel, 3. Social relationships, 4. Emotional health. (31 questions)	77	9.5 (T1: 8.2, T2: 7.1, T3: 13.3)	AT (T1, T2, T3)	9.5
Wagner’s quality of life scale	Questions on daily lives and psychosocial characteristics. (28 questions)	66	9.9	AT	11.8
Self- constructed non-validated questionnaire		28			

ICIQ-UI SF = International Consultation on Incontinence Questionnaire-Urinary Incontinence Short Form, I-OOL = Incontinence Quality of Life, IIQ-7 = Incontinence Impact Questionnaire, N = number, AT = All trimesters, T1 = Trimester 1, T2 = Trimester 2, T3 = Trimester 3

Six different measurement instruments for bother were used, of which the ICIQ-UI SF was most frequently used. Two studies reported the results of the ICIQ-UI SF as categories [25, 26]. One measurement instrument was self-constructed and non-validated [27].

### Synthesis of results

#### Overall prevalence

**Fig. 2:**
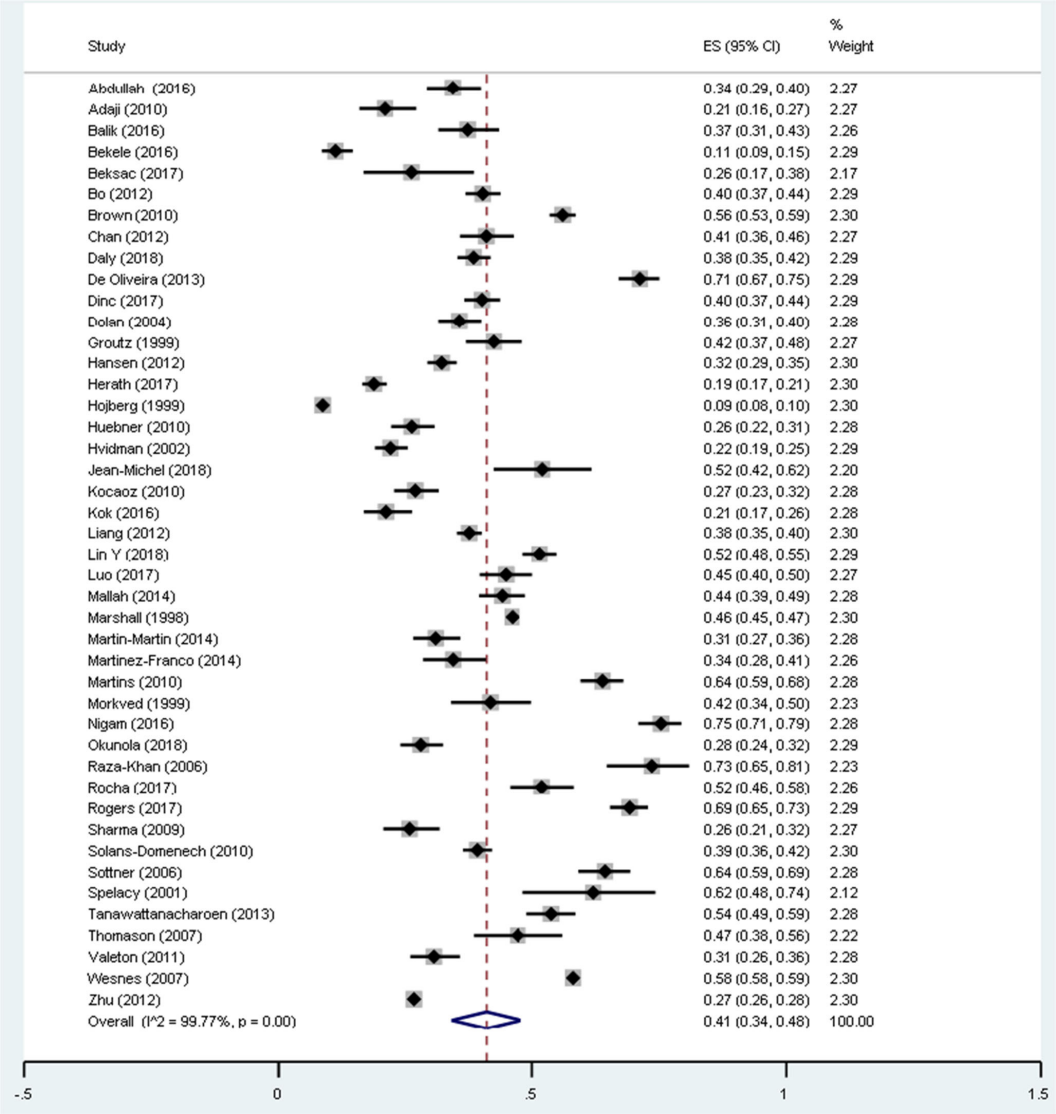
Pooled prevalence of UI during pregnancy

Forty-four studies involving a total of 88.305 women were used to calculate the overall prevalence of UI. The weighted average of UI prevalence among pregnant women was 41.0% (CI 95% 34.0–48.0%; I^2^: 99.77%), regardless of trimester, parity or type of UI (Fig. 2). The lowest prevalence of UI found in the included studies was 9% [28] and the highest prevalence 75% [29]. Prevalence figures for low, moderate and high risk of bias studies were 38% (95% 18.0–58.0), 41% (95% 36.0–46.0) and 47% (95% 39.0–54.0) respectively.

#### Subcategories trimester of pregnancy, type of UI and parity

Five out of the 44 studies included women from trimester 1 or 2 or two out of three pregnancy trimesters. Fifteen studies recruited women from the third trimester, with an overall UI prevalence of 47% (95% CI: 37.0–58.0%). Twenty-four studies recruited women from trimester 1–3, with an overall UI prevalence of 40% (95% CI: 34.0–45.0%). Based on 24 studies, SUI accounts for 63% of UI cases, whereas UUI, MUI and unexplained UI were 12%, 22% and 3% respectively.

When parity is taken into account, 42% of nulliparous women experience UI (based on 12 studies; 95% CI 33.0–51.0%; I^2^ = 98.6%), whereas four studies reporting only on primiparous women found an overall UI prevalence of 31% (95% CI 26.0–36.0%; I^2^ 90.6%). Twenty-seven studies included women with any parity, resulting in a pooled prevalence of 42% (95% CI 32.0–53.0%; I^2^ 99.8%).

Based on 12 out of 44 studies, the overall prevalence for UI in trimesters 1, 2 and 3 is 9% (95% CI 6.0–12.0%; I^2^ 97.7%), 19% (95% CI 12.0–25.0%; I^2^ 98.7%) and 34% (95% CI 23.0–46.0%; I^2^ 99.0%) respectively.

#### Subcategories frequency and amount of UI

Based on ten studies, monthly UI accounts for 40% of UI cases (95% CI 23.0–57.0%; I^2^ 99.0%), weekly UI for 33% (95% CI 23.0–43.0%; I^2^ 94.8%) and daily UI for 26% (95% CI 20.0–32.0%; I^2^ 86.9%).

The majority of studies (*n* = 9), reporting on the amount of urine loss (*n* = 14), used the International Consultation on Incontinence Questionnaire-Urinary Incontinence Short Form (ICIQ-UI SF) to assess this parameter (none, small, moderate, large amount) [30]. Three studies reported separately the ICIQ-UI SF amount item, showing that the majority (79.2–86.9%) of UI cases lose a small amount. Other descriptions of amount of urine lost were: drops or just a little, more like a trickle, more than a trickle [31, 32], a few droplets, a stream [33] and drops, small splashes and more [26, 34].

#### Bother

Thirteen studies reported on impact on daily life, quality of life or bother. It was heterogeneously assessed; however, the ICIQ-UI SF was used in the majority of studies (*n* = 7). In two studies question 3 of the ICIQ-UI SF on interference in daily life was reported as a measurement instrument for bother. Other measurement instruments that were used only once were the Incontinence Quality of Life (I-QOL), Incontinence Impact Questionnaire (IIQ-7), Wagner’s quality of life questionnaire and a self-constructed non-validated questionnaire. The overall bother of UI during pregnancy, on a 0 to 100 scale, ranges between 9.5 and 34.1, consistent with mild to moderate bother, whereas the experienced bother is higher in the 3rd trimester (between 13.3 and 57.6) (Table 3).

**Table 3:** Converted results 0–100 by measurement instrument

	Total (all instruments)	ICIQ-UI SF (total score)	ICIQ-question 3 (QoL)	Other measurement instruments (I-Qol, IIQ-7, Wagner’s QoL)
All trimesters	9.5–34.1 (7 studies)	19.3–31.4 (2 studies)	31.3 (1 study)	9.5–34.1 (3 studies)
3rd trimester	13.3–57.6 (5 studies)	29.5–57.6 (3 studies)	34.8 (1 study)	13.3 (1 study)

ICIQ-UI SF = International Consultation on Incontinence Questionnaire-Urinary Incontinence Short Form, I-QOL = Incontinence Quality of Life, IIQ-7 = Incontinence Impact Questionnaire, wks = weeks

#### Case definition

The majority of studies (*n* = 30) did not specify a case definition for UI. Four studies used as a case definition ‘any leakage’ or used the frequency (*n* = 5), amount/volume (*n* = 1), timeframe (*n* = 2) or UI type (*n* = 3) criteria in their case definition, or a combination of those (*n* = 3).

#### Incidence

Few studies have examined incidence of UI during pregnancy, using different trimesters of pregnancy and case definitions. Therefore, no pooling was done for this outcome. Five studies reported on new-onset UI during pregnancy among women who were continent 12 months before the index pregnancy [31, 32] or who had no UI previous to pregnancy [20, 22, 26]. Daly et al. reported that 21.7% of nulliparous women experienced any new-onset urinary leakage in early pregnancy [32]. The frequency of leakage among new-onset UI was less than once per month in 55% of cases and on a monthly, weekly and daily basis in 26.7%, 13.3% and 5.0% of cases respectively. The majority (83.1%) experienced drops or just a little amount of leakage. Brown et al. [31] reported 146 incident cases for any UI in early pregnancy (≤ 24 weeks of gestation; 16.4%) compared to 561 cases in late pregnancy (31 weeks; 63.2%). It appeared that new cases of SUI accounted for more than two thirds of prevalent cases in early and late pregnancy, 70.4% and 73.9%, respectively. Hvidman et al. concluded that UI incidence during pregnancy was 16.8% among nulliparous and 8.4% among primiparous women [20]. Overall, incidence rates in early pregnancy among nulliparous women range between 16.4% and 21.7% [31, 32]. When considering late pregnancy, the incidence rate increases to 45.6–63.2% [22, 31]. The incidence rate of UI during first pregnancy, regardless of trimester, is 16.8–39.1% [20, 26].

## Discussion

The aim of this systematic review was to examine the pooled prevalence and incidence of UI during pregnancy and to provide an overview of measurement instruments, including the measurement results, to assess bother in relation to UI. The results show an overall mean prevalence of UI during pregnancy of 41%, with a range of 9–75%. The prevalence numbers rise with gestational period from 9% in the first trimester to 34% in the third. SUI is the most prevalent type of UI, accounting for 63% of cases. Twenty-six percent of the women reported daily loss, whereas 40% reported loss on a monthly basis. Most of the cases reported a small amount of urine loss.

Incidence/new onset UI in nulliparous women in early pregnancy varied between 16.4% and 21.7%[31, 32]. This variation might be explained by the different case definition used for UI (e.g. any UI [32] in contrast to UI at least once a month [31]). Incidence in late pregnancy increased to 63.2% [31]. Over 70% of new onset UI was SUI. The high prevalence and rising incidence numbers of SUI during pregnancy might be due to several factors such as physiological weight gain, which results in increased intra-abdominal pressure on the bladder and pelvic floor muscles [35]. Additionally, it is known that pregnant women with SUI have significantly less pelvic floor muscle strength and thickness [36] and/or a larger hiatal area at rest and during pelvic floor muscle contraction [37]. But also previous childbirth and high body mass index are risk factors for developing SUI [38, 39].

Most included studies showed a moderate risk of bias. Although several factors influence reported prevalence rates, e.g. case definition, studies with moderate or high risk of bias may distort prevalence and/or incidence rates. The prevalence rate among three studies with high risk of bias is 47% compared to 38% among studies with low risk of bias (in studies with a moderate risk of bias the prevalence is 41%). As studies with a low risk of bias tend to have a slightly lower prevalence, it is likely that the real prevalence of UI in pregnant women is in the range of 38–41%.

Only 13 out of 44 studies reported bother in relation to UI; these studies used a variety of measurement instruments. In an attempt to provide an overall assessment of experienced bother in relation to UI, we (arbitrarily) chose to standardize the measurement results of different bother scales to a 0 to 100 scale. Bother of UI during pregnancy ranges between 9.5 and 34.1 on a (standardized) 0 to 100 scale. The 0 to 100 scale can be regarded as a visual analogue scale (VAS). The VAS is a valid and reproducible method to measure the impact of UI on QOL [40]. No studies are known that report on cut-off points for QOL in pregnant women with UI. Therefore, cut-off points must be interpreted cautiously. One study comparing the VAS with a measure that assesses the impact on functioning in patients with pain identified three classes: class 1, mild interference (score 1–34); class 2, moderate interference (score 35–64); class 3, severe interference with daily life (score 65–100) [41]. Based on these classes the overall bother of UI during pregnancy is mild and in the third trimester mild to moderate. One study reporting on bother of UI in the last 4 weeks of pregnancy reported the highest bother of 57 [42]. This might be due to the rising prevalence over time in pregnancy [29, 34, 42–46].

The ICI provides an overview of (recommended) grade A (high-quality) measurement instruments for bother in relation to UI [3] and advises to report prevalence figures in combination with the experienced bother. The ICIQ-UI SF, IIQ and I-QOL, for example, are rated as grade A measurement instruments. Wagner’s QOL and the VAS are not incorporated in the ICI overview, nor is the separate use of question 3 of the ICIQ-UI SF as a bother measure. A closer look at the measurement instruments shows that there are differences with regard to assessed constructs and domains. The ICIQ-UI SF is a quick way to assess frequency, severity and bother of UI. The IIQ, I-QOL and Wagner’s QOL scale assess bother of UI with a variety of subscales such as psychosocial impact, social embarrassment, relations, and physical activity and provide therefore more in-depth information.

This systematic review revealed that the reporting of prevalence with a measure of bother is not common practice yet. To improve the reporting of UI prevalence, it is recommended that in research projects both prevalence and bother should be measured with high quality measurement instruments in line with the recommendations of ICI. In clinical practice, measurement results of bother support healthcare professionals in the clinical reasoning process as it may provide information on diagnosis or prognosis or may evaluate one’s own actions. At the same time, it standardizes communication with colleagues. Moreover, measurement results can be used to better inform patients about their situation and to involve them more easily in joint therapy decisions (shared decision making).

The construct bother in relation to UI seems difficult to grasp, as included studies used different definitions. The following terms were used: effect on daily activities/everyday life, interference on daily life, health-related quality of life, severity, lifestyle changes, (perceived) impact on quality of life, distress, experienced discomfort and amount of bother. As the degree of bother is related to help-seeking behaviour for UI [47, 48], it is of importance to define the construct bother (what does bother in relation to UI mean for pregnant women) and quantify bother. When bother is well defined and quantified, this will support researchers in selecting the appropriate measurement instrument and interpretation of the results.

Based on the prevalence figures, it would appear that UI in pregnancy is an enormous healthcare problem. However, not everyone seeks (medical) help for UI immediately. Several factors determine help-seeking behaviour of pregnant women, such as awareness of treatment possibilities and the experienced burden of UI [48, 49]. Also the belief that UI will resolve by itself after delivery and the lack of knowledge that UI during pregnancy raises the odds for post-partum UI substantially obstructs help-seeking [50, 51].

Management of UI should be directed to women who seek help for UI, but may also be directed towards women who experience bother or have risk factors for developing UI (prevention). Such uncertainties require further evaluation and data on duration of treatment effects of PFM(G)T [52]. Maternity care workers need to assess women for the presence, severity and bother of UI and, in consultation with them, develop a specially tailored plan of care to meet the women’s needs.

The strength of this systematic review is the large number of included studies, which resulted in the availability of prevalence and incidence numbers for different subpopulations (countries, parity, trimester of pregnancy) and for different purposes (research planning, health care providers and policy makers). To our knowledge, this review is the first one that focused on assessment methods for bother in relation to UI and degree of adherence to the recommendations of ICI with regard to this topic.

The limitations of this systematic review are, first, the presence of substantial clinical heterogeneity of the studies. Clinical heterogeneity is due to differences in: case definition (any UI or different frequencies of UI in a certain period of time), population (primigravida-multigravida) or periods researched (first, second, third trimester or any specific trimester). Second, the considerable statistical heterogeneity of the studies resulted in large CIs. Third, as the Joanna Briggs critical appraisal tool does not recommend cut-off points for high, moderate or low risk of bias, we arbitrarily chose the cut-off points reported in this systematic review to explore possible differences in prevalence numbers if stratified for risk of bias. However, we did not include or exclude studies based on risk of bias.

## Conclusion

UI is a very common symptom in pregnancy, and the prevalence rises as weeks of gestation progress. SUI is the most common types and in most of the cases a small amount of urine was lost. The level of bother for UI is heterogeneously assessed and is experienced as mild to moderate by pregnant women.

## Appendix

Search strategy for PubMed:

(((((((((((((((((((pregnancy[MeSH Terms]) OR pregnancy[Title/Abstract]) OR pregn*) OR prepartum[Title/Abstract]) OR ‘pre-partum’[Title/Abstract]) OR ‘pre partum’[Title/Abstract]) OR peripartum[Title/Abstract]) OR ‘peri-partum’[Title/Abstract]) OR ‘peri partum’[Title/Abstract]) OR nulliparous[Title/Abstract]) OR primiparous[Title/Abstract]) OR primigrav*[Title/Abstract]) OR primipar*[Title/Abstract]) OR multiparous[Title/Abstract]) OR multigrav*[Title/Abstract]) OR multipar*[Title/Abstract])) AND (((((((((((‘urinary incontinence’[MeSH Terms]) OR urinary incontinence title/abstract) OR ‘urine loss’[Title/Abstract]) OR ‘pelvic floor disorders’[MeSH Terms]) OR ‘pelvic floor disorders’[Title/Abstract]) OR ‘pelvic floor dysfunctions’[Title/Abstract])) OR incontinence[Title/Abstract])) OR ‘leaking urine’[Title/Abstract]))) AND ((((((((((((prevalence[MeSH Terms]) OR prevalence[Title/Abstract]) OR epidemiology[MeSH Terms]) OR epidemiology[Title/Abstract]) OR quality of life[MeSH Terms]) OR ‘quality of life’[Title/Abstract]) OR bother*[Title/Abstract]) OR bothersomeness[Title/Abstract]))))))
